# Variation in GP decisions on antihypertensive treatment in oldest-old and frail individuals across 29 countries

**DOI:** 10.1186/s12877-017-0486-4

**Published:** 2017-04-20

**Authors:** Sven Streit, Marjolein Verschoor, Nicolas Rodondi, Daiana Bonfim, Robert A. Burman, Claire Collins, Gerasimovska Kitanovska Biljana, Sandra Gintere, Raquel Gómez Bravo, Kathryn Hoffmann, Claudia Iftode, Kasper L. Johansen, Ngaire Kerse, Tuomas H. Koskela, Sanda Kreitmayer Peštić, Donata Kurpas, Christian D. Mallen, Hubert Maisoneuve, Christoph Merlo, Yolanda Mueller, Christiane Muth, Marija Petek Šter, Ferdinando Petrazzuoli, Thomas Rosemann, Martin Sattler, Zuzana Švadlenková, Athina Tatsioni, Hans Thulesius, Victoria Tkachenko, Peter Torzsa, Rosy Tsopra, Tuz Canan, Rita P. A. Viegas, Shlomo Vinker, Margot W. M. de Waal, Andreas Zeller, Jacobijn Gussekloo, Rosalinde K. E. Poortvliet

**Affiliations:** 1 Institute of Primary Health Care (BIHAM), University of Bern, Bern, Switzerland; 2 Department of General Internal Medicine, Inselspital, Bern University Hospital, University of Bern, Bern, Switzerland; 3 Hospital Israelita Albert Einstein, São Paulo, Brazil; 4 Vennesla Primary Health Care Centre, Bergen, Norway; 5 Irish College of General Practitioners, Dublin, Ireland; 6 Department of Nephrology and Department of Family Medicine, University Clinical Centre, University St. Cyril and Metodius, Skopje, Macedonia; 7 Faculty of Medicine, Department of Family Medicine, Riga Stradiņs University, Riga, Latvia; 8 Institute for Health and Behaviour, Research Unit INSIDE, University of Luxembourg, Luxembourg, Luxembourg; 9 Department of General Practice and Family Medicine, Center for Public Health, Medical University of Vienna, Vienna, Austria; 10 Timis Society of Family Medicine, Sano Med West Private Clinic, Timisoara, Romania; 11 Danish College of General Practitioners, Copenhagen, Denmark; 12 School of Population Health, University of Auckland, Auckland, New Zealand; 13 Department of General Practice, University of Tampere, Tampere, Finland; 14 Family Medicine Department, Health Center Tuzla, Medical School, University of Tuzla, Tuzla, Bosnia and Herzegovina; 15 Family Medicine Department, Wroclaw Medical University, Wrocław, Poland; 16 Primary Care and Health Sciences, Keele University, Keele, Staffordshire ST5 5BG UK; 17 Primary Care Unit, Faculty of Medicine, University of Geneva, Geneva, Switzerland; 18 Institute of Primary and Community Care Lucerne (IHAM), Lucerne, Switzerland; 19 Institute of Family Medicine Lausanne (IUMF), Lausanne, Switzerland; 20 Institute of General Practice, Goethe-University, Frankfurt / Main, Germany; 21 Department for Family Medicine, Medical faculty, University of Ljubljana, Ljubljana, Slovenia; 22 SNAMID (National Society of Medical Education in General Practice), Prata Sannita, Italy; 23 Department of Clinical Sciences in Malmö, Centre for Primary Health Care Research, Lund University, Malmö, Sweden; 24 Institute of Primary Care, University Hospital Zurich, University of Zurich, Zurich, Switzerland; 25 SSLMG, Societé Scientifique Luxembourgois en Medicine generale, Luxembourg, Luxembourg; 26 Ordinace Řepy, s.r.o., Prague, Prague, Czech Republic; 27 Research Unit for General Medicine and Primary Health Care, Faculty of Medicine, School of Health Sciences, University of Ioannina, Ioannina, Greece; 28 Family Medicine, Department of Clinical Sciences, Lund University, Malmö and senior researcher Region Kronoberg, Växjö, Sweden; 29 Department of Family Medicine, Institute of Family Medicine at Shupyk National Medical Academy of Postgraduate Education, Kiev, Ukraine; 30 Department of Family Medicine, Semmelweis University, Budapest, Hungary; 31 LIMICS, INSERM, U1142, F-75006 Paris, Université Paris 13, Sorbonne Paris Cité, UMR_S 1142, F93000 Bobigny, Sorbonne Universités, UPMC Université Paris 06, UMR_S 1142, F75006 Paris, Paris, France; 32 Leeds Centre for Respiratory Medicine, St James’s University Hospital, Beckett Street, Leeds, LS9 7TF UK; 33 Family Medicine Specialist, Kemaliye Town Hospital, Erzincan University, Erzincan, Turkey; 34 Family Doctor, Invited Assistant of the Department of Family Medicine, NOVA Medical School, Lisbon, Portugal; 35 Sackler Faculty of Medicine, Tel Aviv University, Tel Aviv, Israel; 36 Centre for Primary Health Care (uniham-bb), Basel, Switzerland; 37 Department of Public Health and Primary Care, Leiden University Medical Center, Hippocratespad 21, 2333 ZD Leiden, The Netherlands

**Keywords:** Hypertension, Oldest-old, Clinical variation, General practitioners, Frailty, Elderly

## Abstract

**Background:**

In oldest-old patients (>80), few trials showed efficacy of treating hypertension and they included mostly the healthiest elderly. The resulting lack of knowledge has led to inconsistent guidelines, mainly based on systolic blood pressure (SBP), cardiovascular disease (CVD) but not on frailty despite the high prevalence in oldest-old. This may lead to variation how General Practitioners (GPs) treat hypertension. Our aim was to investigate treatment variation of GPs in oldest-olds across countries and to identify the role of frailty in that decision.

**Methods:**

Using a survey, we compared treatment decisions in cases of oldest-old varying in SBP, CVD, and frailty. GPs were asked if they would start antihypertensive treatment in each case. In 2016, we invited GPs in Europe, Brazil, Israel, and New Zealand. We compared the percentage of cases that would be treated per countries. A logistic mixed-effects model was used to derive odds ratio (OR) for frailty with 95% confidence intervals (CI), adjusted for SBP, CVD, and GP characteristics (sex, location and prevalence of oldest-old per GP office, and years of experience). The mixed-effects model was used to account for the multiple assessments per GP.

**Results:**

The 29 countries yielded 2543 participating GPs: 52% were female, 51% located in a city, 71% reported a high prevalence of oldest-old in their offices, 38% and had >20 years of experience. Across countries, considerable variation was found in the decision to start antihypertensive treatment in the oldest-old ranging from 34 to 88%. In 24/29 (83%) countries, frailty was associated with GPs’ decision not to start treatment even after adjustment for SBP, CVD, and GP characteristics (OR 0.53, 95%CI 0.48–0.59; ORs per country 0.11–1.78).

**Conclusions:**

Across countries, we found considerable variation in starting antihypertensive medication in oldest-old. The frail oldest-old had an odds ratio of 0.53 of receiving antihypertensive treatment. Future hypertension trials should also include frail patients to acquire evidence on the efficacy of antihypertensive treatment in oldest-old patients with frailty, with the aim to get evidence-based data for clinical decision-making.

**Electronic supplementary material:**

The online version of this article (doi:10.1186/s12877-017-0486-4) contains supplementary material, which is available to authorized users.

## Background

Hypertension is the most important preventable cause of poor cardiovascular outcome and is responsible for disability and deaths from stroke, myocardial infarction and other diseases [1]. Treating hypertension is beneficial and (since the 1990s) it is known that treatment also reduces stroke rates and myocardial infarction in patients aged >60 years [2–4]. As life expectancy has increased worldwide, a new term was needed to describe those in the fastest-growing age group expected to triple within the next 35 years [5], i.e. the group ‘oldest-old’ is now defined as those aged >80 years.

The population of the oldest-old is heterogeneous. Some oldest-old are very healthy whereas others are multimorbid with complex problems. Although the group of multimorbid oldest-old is rapidly increasing, most trials still exclude them. Messerli et al. highlighted this commonly-applied exclusion by applying exclusion criteria taken from 13 hypertension trials with oldest-old participants, to a primary care cohort of hypertensive patients aged >60 years [6]: in this case, ≥70% of the oldest-old would have been excluded and they were both older and sicker.

The exclusion of such a large percentage of oldest-old has caused a serious gap in our knowledge and in guidelines to treat hypertension in patients with multimorbidity. Even more scarce are recommendations for frail patients: for example, of six current hypertension guidelines, only those of the European Society of Hypertension and of the European Society of Cardiology have a specific recommendation to leave decisions on antihypertensive therapy in the frail and oldest-old patients to the treating physician (class I C recommendation) [7].

Due to the current lack of clear evidence, the best management of hypertension in the oldest-old remains unknown; this may, in turn, lead to clinical variation. Although it is difficult to quantify, variation exists in the way that the best available evidence is applied in clinical practice [8]. Among the diverse reasons for this variation, the appropriateness of guidelines for physicians in treating specific groups of patients is of particular importance. However, to reduce clinical variation and improve quality of care/patient safety, there is a need to assess clinical variation among the oldest-old patients, who are consistently excluded from trials but suffer from both multimorbidity and frailty.

Therefore, the present study investigates clinical variation across countries of general practitioners’ (GPs) decisions to start antihypertensive treatment in patients aged >80 years. Our hypothesis was that *frailty* would be an important factor in deciding *not* to start antihypertensive treatment in clinical practice, although this is not specifically addressed in most guidelines.

## Methods

### Design

GPs from different countries were invited to participate in a survey based on case vignettes.

### Setting

The aim was to recruit national representatives (defined as a GP in contact with a national GP network) of 40 countries on the European continent, and in Brazil and New Zealand. We also re-contacted six national representatives of GP networks participating in a previous survey [9]. Also invited to participate were: 1) national representatives of WONCA Europe (European Branch of the World Organization of National Colleges, Academies and Academic Associations of General Practitioners/Family Physicians) [10]; 2) the European General Practice Research Network (EGPRN) [11]; and 3) the Network of Junior GPs in Europe (the Vasco da Gama Movement, VdGM) [12].

The study was conducted in accordance with the Declaration of Helsinki [13]. Because the responses of GPs were collected anonymously, most countries required no approval from an ethics committee. In countries where approval was mandatory (Switzerland, Brazil), a waiver from the ethics committee was obtained. In New Zealand, approval for the study was granted by the University of Aukland Ethics Committee.

### Participants

All national representatives were asked to include as many GPs as possible from their GP network. Because primary care surveys usually score low on response rates, we regularly reported the numbers of participating GPs to the national coordinators, so they could send reminders if needed. The only inclusion criterium for the survey was to be actively working as a GP; this was asked at the beginning of the survey. Participants who did not meet this criterium (e.g. due to retirement) were excluded from completing the survey.

### Procedures

Beforehand, we developed/tested the survey for optimal technicality between SurveyMonkey (www.surveymonkey.com, Palo Alto, CA, USA) and Stata, among five GPs. Then, to test for clarity/feasibility, the survey was piloted among a sample of 16 physicians working in Switzerland.

National representatives translated the survey from English to their own language. Finally, the survey was available in 21 languages. National representatives of Greece, Israel and Finland decided to distribute the survey in English. The correctness of all translations was evaluated by the team of collaborators.

The survey can be accessed online (see Additional file 1). First, we asked the GP’s gender, office location (city, suburban, rural), and years of experience working as a GP (in 5-years bands). Second, GPs were asked to estimate the proportions of patients aged >80 years attending their GP office. Third, eight case vignettes were presented of oldest-old patients of both gender, presenting for a routine visit in a GP office without blood pressure-related symptoms and not receiving any antihypertensive treatment. For each case vignette, GPs were asked to decide if they would start antihypertensive treatment. All case vignettes differed in three primary characteristics: systolic blood pressure (SBP), cardiovascular disease (CVD), and frailty (see Additional file 2). SBP was either 140 mmHg or 160 mmHg. CVD was either present (e.g. case vignettes with a history of myocardial infarction or stroke) or absent. Because the condition of frailty lacks a common definition [14], we stated that frailty is defined as patients with at least two of the following criteria: unintentional weight loss, exhaustion, low level of activity, muscle weakness, and slow gait speed. Thus, a patient with a low level of activity and unintentional weight loss was considered to be frail. To facilitate filling in the survey, for each case vignette we indicated one of the following statements: “*You consider this patient to be frail*” or “*You don’t consider this patient to be frail*”.

The survey was distributed by email between March 9 and July 31 2016. As the only exception, Ukraine distributed the survey on paper during a regional GP meeting because there is insufficient internet access for GPs in Ukraine.

### Statistical analysis

To describe baseline characteristics, proportions were calculated for dichotomized or categorized data, and means were calculated for continuous data.

To assess international variation in decisions for treatment, per country the crude proportions and confidence intervals (CI) were calculated for GPs who would start treatment.

To assess the role of frailty in the decision to start treatment per country, odds ratios (ORs) and CI were calculated per country using a mixed-effects model adjusted for GP’s gender, years of experience, office location, prevalence of oldest-old in the GP practice, guideline compliance, SBP, and CVD. The mixed-effects model was used to account for the multiple assessments per GP. The estimate of each country was presented on a forest plot.

For each case vignette, we calculated the crude proportions of GPs starting treatment and also compared two corresponding case vignettes (e.g. in Case 1 the patient is not frail, whereas in Case 2 the patient is frail).

To assess the overall influence of SBP, CVD and frailty, the same mixed-effects model was used but, in addition, clustering within countries was taken into account.

A two-sided *p*-value of 0.05 was considered statistically significant. Analyses were performed with STATA 14.2 (StataCorp, College Station, TX, USA).

## Results

From March through July 2016, we contacted 40 national representatives from Europe, Brazil, Israel, Russia, and New Zealand and received replies from 29 countries. Overall, 13,671 GPs were invited, of whom 2585 responded. Subsequently, 42 respondents were excluded because they were no longer working as a GP, resulting in 2543 participants. The median response rate was 26% (IQR 10–62%) (see Additional file 3).

Table 1 presents the baseline characteristics of the participating GPs; 52.3% were female, 50.8% lived in a city, and 37.6% had >20 years of experience. The majority of GPs (61.3%) estimated the prevalence of the oldest-old patients in their practice to be >10%.

**Table 1:** Baseline characteristics of participating GPs from 29 countries

Baseline characteristics (*N* = 2543)	*n* (%)
Female GP	1341 (52.3)
Practice location	
City	1292 (50.8)
Suburban	599 (23.6)
Rural	651 (25.6)
Experience as GP	
< 5 years	471 (18.5)
5–10 years	445 (17.5)
11–15 years	341 (13.4)
16–20 years	328 (12.9)
> 20 years	956 (37.6)
Self-estimated prevalence of patients >80 years at own practice	
< 10%	851 (38.7)
10–20%	865 (39.4)
21–30%	323 (14.7)
> 30%	159 (7.2)

**Fig. 1:**
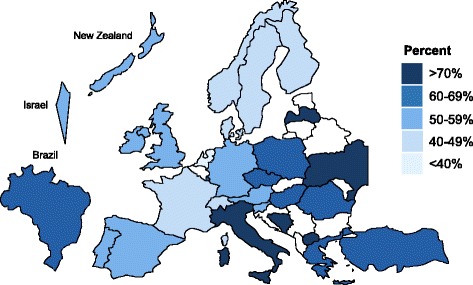
National percentages in which general practitioners decide to start antihypertensive treatment in all eight cases of oldest-old patients (unadjusted)

Overall, the crude proportions of treatment varied considerably between countries (Fig. 1). For example, the lowest proportion of treatment was found in the Netherlands (34.2%; 95% CI 32.0–36.5%) whereas Ukraine had the highest proportion (88.3%; 95% CI 85.3–90.9%).

**Fig. 2:**
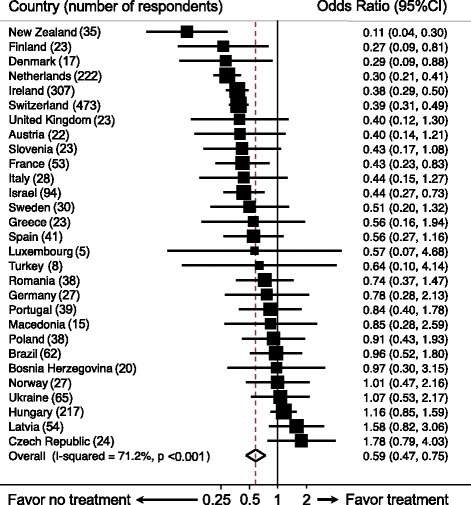
Influence of frailty on 2053 general practitioners (GPs) when deciding to start antihypertensive treatment per country (adjusted^a^). ^a^Adjusted for GP characteristics (gender, experience, location, prevalence of oldest-old, guideline compliance) and patient characteristics (cardiovascular disease, systolic blood pressure). A mixed-effects model was used to account for multiple assessments per GP. Although 2543 GPs participated, missing data on GPs’ decisions to treat the eight cases means that only 2053 GPs are included here

Figure 2 shows the GPs’ treatment probability in frail oldest-old compared to non-frail oldest-old for each of the 29 countries. Overall, the treatment probability for all countries was OR 0.59 (95% CI 0.47–0.75) and the probability per country ranged from OR 0.11 in New Zealand to 1.78 in the Czech Republic. In 8/29 (28%) countries (i.e. New Zealand, Finland, Denmark, the Netherlands, Ireland, Switzerland, France and Israel) we are 95% confident that GPs would be less likely to start antihypertensive treatment in the frail oldest-old patients compared to the non-frail oldest-old patients. In 16/29 (55%) countries, an OR <1 was found but a 95% CI including 1; this larger 95% CI was due to the lower number of respondents per country (<30 per country in 45% of all countries). In 5/29 (17%) countries, the OR was >1 but (to a large extent) the 95% CI included 1.

GPs’ decision to treat hypertension in the oldest-old varied considerably, ranging from 17.3% to 96.8% according to the specific case vignette (Table 2). The lowest level of treatment decision was scored in those case vignettes that included no frailty, no CVD, and a SBP 140 mmHg (17.3%; 95% CI 15.7–19.0%). The case vignettes that included CVD, SBP 160 mmHg and no frailty scored the highest (96.8%; 95% CI 95.9–97.5%). Besides frailty (adjusted OR 0.53; 95% CI 0.48–0.59), a SBP of 140 mmHg (adjusted OR 0.01; 95% CI 0.01–0.01) and no CVD (adjusted OR 0.29; 95% CI 0.26–0.32) were also independent factors that caused GPs not to start treatment.

**Table 2:** Percentages of general practitioners (GPs) starting antihypertensive treatment for the eight individual cases (*n* = 2053 GPs)

Cases	Proportion of GPs starting treatment	Case Characteristics		
	% (95% CI)	Frailty	CVD	SBP 160 mmHg
Overall	54.9 (54.1–55.7)			
Case 1	17.3 (15.7–19.0)	−	−	−
Case 2	18.2 (16.6–20.0)	+	−	−
Case 3	85.4 (83.7–86.9)	−	−	+
Case 4	75.6 (73.6–77.5)	+	−	+
Case 5	96.8 (95.9–97.5)	−	+	+
Case 6	84.9 (83.2–86.4)	+	+	+
Case 7	32.5 (30.4–34.6)	−	+	−
Case 8	29.5 (27.5–31.6)	+	+	−

*CVD* cardiovascular disease, *SBP* systolic blood pressure

Although 2543 GPs participated, missing data on GPs’ decisions to treat the eight cases means that only 2053 GPs are included here

## Discussion

After sampling >2500 GPs in 29 countries, this study revealed large clinical variation in starting antihypertensive treatment (ranging from 34 to 88%) based on case vignettes of oldest-old patients. As hypothesized, frailty proved to be an important patient characteristic for GPs in deciding whether or not to start antihypertensive treatment in 24/29 (83%) countries. The probability of a GP treating a frail patient was almost half that compared with a GP managing a non-frail patient. Current guidelines are clearer about the level of SBP related to initiating treatment; this was confirmed in the present study in which GPs were less inclined to start treatment in the case of SBP 140 mmHg compared to SBP 160 mmHg. Nevertheless, how to manage frailty will become increasingly important for an increasingly older and multimorbid population. When specific data from future trials that include frail patients become available, hypertension and other guidelines can be updated accordingly.

### Scientific and clinical context of the results

Treatment goals for hypertension are constantly changing [15]. Recent trials including oldest-old patients indicate aiming at the lower levels of SBP [3, 16]. However, these latter patients may differ from the general population that GPs are managing, due to the extensively applied exclusion criteria for the older and sicker patients [6]. Therefore, it remains unclear whether lowering SBP in multimorbid and frail patients does in fact lead to better outcomes. For example, in the SPRINT trial, frail patients showed smaller intertreatment group differences in SBP compared to non-frail patients, thus a lower SBP might be harder to achieve in frail patients [16]. On the other hand, there is evidence that frail oldest-old need a higher SBP. In a recent meta-analysis comparing pro- and retrospective cohort studies, Zhang et al. found that a higher SBP in frail oldest-old patients had a protective effect in lowering the risk of overall mortality [17]. Thus, current knowledge seems to be well summarized by Materson et al. who suggested to evaluate and treat frail oldest-old patients individually, while the healthier oldest-old should be treated regardless of their chronological age [18].

In the present study, this wide spectrum of recommendations and lack of clear evidence may partly explain the variation found between the participating countries. Differences in national guidelines/campaigns may have also led to differences between the countries. Nevertheless, this study confirmed our hypothesis that frailty is a factor that GPs take into consideration when starting antihypertensive treatment; moreover, we found that GPs were less likely to treat frail patients, even after adjusting for SBP and CVD. This is in line with findings from a Dutch qualitative study, where vulnerability was an important patient-related barrier for GPs when implementing guidelines for secondary cardiovascular prevention in oldest-old [19].

Interestingly, our findings share some findings and yet show difference with the only other published study on this topic. Mermans et al. conducted a similar survey among 305 GPs in Belgium. These authors also found large differences in treatment intentions for hypertension in the oldest-old patients between GPs and showed that there was a significant difference in the treatment intention of GPs between robust patients and strongly dependent patients. However, the stated that ‘differences in the patients’ level of dependency were not responsible for the variation in the overall treatment intention’ [20]. However, on an international level, when including many countries, frailty was established as an important factor influencing GPs’ treatment decisions.

### Strengths and limitations

A strength of this study is the high number of countries and relatively large number of respondents (thanks to collaboration with WONCA Europe, EGPRN, and VdGM). Further, the sampled GPs were experienced with treating oldest-old patients. The inclusion of many countries enabled to produce a detailed map of treatment decision-making in Europe and elsewhere. In addition, we could establish that, in most countries, frailty is associated with a lower intention to treat, even when taking SBP and cardiovascular comorbidity into account.

This study has several limitations. First, although we report what the GPs stated they would do, this is not necessarily the same as what they would actually do. However, given the realistic case descriptions and the anonymous nature of the survey, we are relatively confident that this limitation has not introduced a systematic bias. Second, the response rate varied considerably between countries and the median rate was only 26%; this is a commonly occurring problem in primary care surveys [21]. However, our response rate was well within the range of other published survey among GPs in major journals [22]. Several reviews further noted that a low response rates in GP survey do not necessarily introduce selections bias [23, 24]. Third, in the case vignettes, only three patient characteristics were taken into consideration. However, because we focused on variation in treatment decision and the role of frailty in that decision, it was beyond the scope of this study to address all possible reasons related to GPs’ treatment decision-making. Fourth, we mainly recruited one GP network per country, which is a selection of GPs dependent on their region of origin or area of interest; however, by adjusting our analysis for GP characteristics we aimed to take this possible confounder into account.

### Implications

This study has several implications for research and clinical practice. First, the large variation in starting treatment in hypertensive oldest-old calls for high-quality cohort studies or (ideally) new hypertension trials specifically including frail patients to acquire evidence as to whether frailty is indeed an important factor when treating hypertension in oldest-old patients. Second, future studies should investigate whether treatment variation might be explained by e.g. the recommendations in guidelines that individual GPs follow. Third, qualitative studies could help us to understand more of the variation we have found. If reasons for the international variation in treatment are established, educational campaigns can be launched to unify the quality of care in Europe (and elsewhere) based on the current body of evidence. Finally, future hypertension guidelines should stratify their recommendations not only for age, blood pressure level and cardiovascular comorbidity, but also for frailty.

## Conclusions

In Europe, Brazil, Israel and New Zealand, GPs’ decisions concerning starting antihypertensive treatment in the oldest-old varied considerably. Independently, the frail oldest-old patients had an almost 50% lower probability for their GP to consider them eligible to receive antihypertensive treatment. Future hypertension trials should also include frail patients to acquire evidence on the efficacy of antihypertensive treatment in oldest-old patients with frailty, with the aim to support and unify clinical decision-making.

## Additional files

Additional file 1: Survey. (DOCX 49 kb)

Additional file 2: Characteristics of the eight case vignettes used in this survey. (DOCX 18 kb)

Additional file 3: Participating countries: number of invited GPs and response rates per country. (DOCX 21 kb)

## Abbreviations

CI: Confidence interval
CVD: Cardiovascular disease
EGPRN: European General Practice Research Network
GP: General practitioner
IQR: Interquartile range
OR: Odds ratio
SBP: Systolic blood pressure
VdGM: The Vasco da Gama Movement
WONCA Europe: European Branch of the World Organization of National Colleges, Academies and Academic Associations of General Practitioners/Family Physicians
