# Supplementary material for: Variation in GP decisions on antihypertensive treatment in oldest-old and frail individuals across 29 countries
Source: BMC Geriatr. 2017 Apr 20;17:93. doi: 10.1186/s12877-017-0486-4 (PMC5399328; doi:10.1186/s12877-017-0486-4)
Supplement: Supplementary file 1 — Survey. (DOCX 49 kb) [file 12877_2017_486_MOESM1_ESM.docx]

**Additional file 1 – Survey**

**Antihypertensive treatment variation in very elderly
Welcome**

**Dear colleague,**

**We welcome you to our international survey that investigates the decisions of general practitioners (GPs) when considering treating very elderly patients (>80 years) for hypertension.**

**This study is conducted by two Institutes of Primary Care in Leiden (the Netherlands) and in Bern (Switzerland).**

**We estimate 5-10 minutes are required to complete this survey.**

**All your answers are collected and treated anonymously.**

**With kind regards,**

**Jacobijn Gussekloo and Sven Streit**

*1. Are you currently working as a General Practitioner?


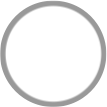
 Yes


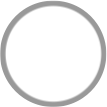
 No (will result in exclusion from this study)

**Antihypertensive treatment variation in very elderly**

Basic characteristics

* 2. What is your gender?


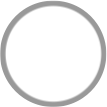
 Female
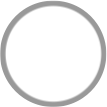
 Male

* 3. In what type of area do you practice family medicine?


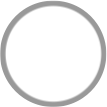
 City


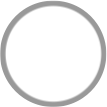
 Suburban


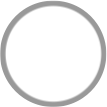
 Rural

* 4. How many years have you been working as a General Practitioner?


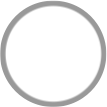
 <5


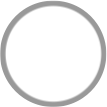
 5-10


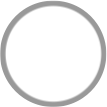
 11-15


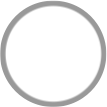
 16-20


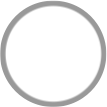
 >20

**Antihypertensive treatment variation in very elderly**Treating patients aged >80 years

- * 5. What percentage of patients in your practice is aged 80 years or older?


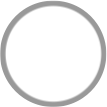
 <10


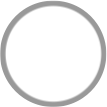
 10-20


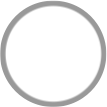
 21-30

-
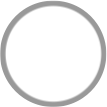
 >30
- * 6. What ideal range do you aim for systolic blood pressure in patients aged >80 years?
- upper limit
- lower limit
- * 7. Is your treatment goal based on national or international guidelines?


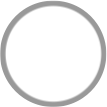
 Yes

-
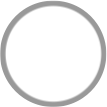
 Mainly yes


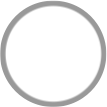
 neutral

-
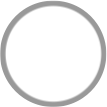
 Mainly no
-
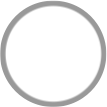
 No
- * 8. Please name the Guideline you consult when treating hypertension in patients aged >80 years?

**Antihypertensive treatment variation in very elderly**

**Cases**

**You will be presented with 8 different cases and are requested to decide whether or not, for each case, you would start antihypertensive treatment.**

**Each case involves a very elderly patient (>80 years) presenting at your GP office for a routine control. None of the patients has blood pressure-related complaints and none is receiving any type of antihypertensive treatment.**

**Although we give only minimal information about each of these cases, this is done purposefully in order to focus on a selected subset of patient characteristics.**

**When we use the term ‘frail’, we define this as patients with at least 2 of the following 5 characteristics: unintentional weight loss, exhaustion, low level of activity, muscle weakness and slow gait speed.**

**Thank you for giving us your best response to each of these cases.**

**Antihypertensive treatment variation in very elderly**Case 1

* 9. 
**Male patient, 82 years, independent living with spouse, some help in housekeeping**

**Findings**
- Systolic blood pressure 140 mmHg
- Unintentional weight loss 
- Diabetes

**Your assessment**- Preventive strategy for hypertension is advised according to current guidelines
- You don't consider this patient to be frail

**Do you start antihypertensive treatment in this patient?**


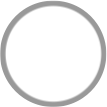
 Yes
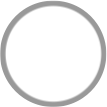
 No

* 10. **What would be your ideal treatment goal for this patient’s systolic blood pressure (in mmHg)?**


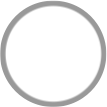
 <120
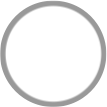
 141-150
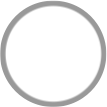
 >170

-
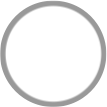
 120-130
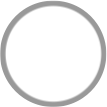
 151-160


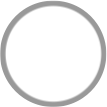
 131-140
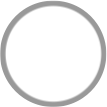
 161-170

**Antihypertensive treatment variation in very elderly**Case 2

*** 11. 
Male patient, 83 years, independent living with spouse, some help in
housekeeping**

**Findings**
- Systolic blood pressure 140 mmHg
- Unintentional weight loss 
- Slow gait speed
- Diabetes

**Your assessment**- Preventive strategy for hypertension is advised according to current guidelines
- You consider this patient to be frail

**Do you start antihypertensive treatment in this patient?**


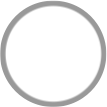
 Yes
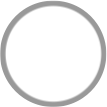
 No

* 12. **What would be your ideal treatment goal for this patient’s systolic blood pressure (in mmHg)?**


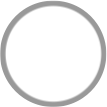
 <120
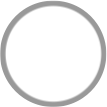
 141-150
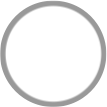
 >170

-
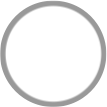
 120-130
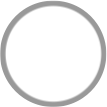
 151-160


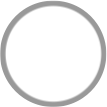
 131-140
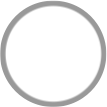
 161-170

**Antihypertensive treatment variation in very elderly**Case 3

*** 13. 
Female patient, 82 years, living in a residence with spouse, some help in housekeeping**

**Findings**
- Systolic blood pressure 160 mmHg
- Slow gait speed
- Obesity
- Hypercholesterolemia

**Your assessment**- Preventive strategy for hypertension is advised according to current guidelines
- You don’t consider this patient to be frail

**Do you start antihypertensive treatment in this patient?**


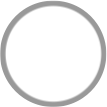
 Yes
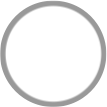
 No

* 14. **What would be your ideal treatment goal for this patient’s systolic blood pressure (in mmHg)?**


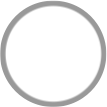
 <120
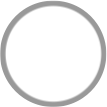
 141-150
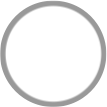
 >170

-
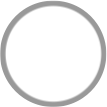
 120-130
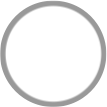
 151-160


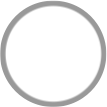
 131-140
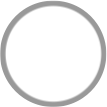
 161-170

**Antihypertensive treatment variation in very elderly**Case 4

*** 15. 
Female patient, 82 years, living alone in a residence, some help in housekeeping**

**Findings**
- Systolic blood pressure 160 mmHg
- Low level of activity
- Slow gait speed  
- Diabetes

**Your assessment**- Preventive strategy for hypertension is advised according to current guidelines
- You consider this patient to be frail

**Do you start antihypertensive treatment in this patient?**


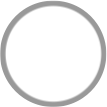
 Yes
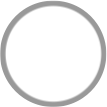
 No

* 16. **What would be your ideal treatment goal for this patient’s systolic blood pressure (in mmHg)?**


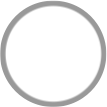
 <120
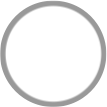
 141-150
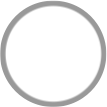
 >170

-
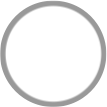
 120-130
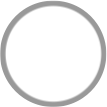
 151-160


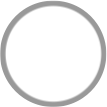
 131-140
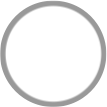
 161-170

**Antihypertensive treatment variation in very elderly**Case 5

*** 17. 
Male patient, 82 years, independent living with spouse, some help in housekeeping**

**Findings**
- Systolic blood pressure 160 mmHg
- Low level of activity
- Myocardial infarction 2 years ago

**Your assessment**- Secondary preventive strategy for hypertension is advised according to current guidelines
- You don't consider this patient to be frail

**Do you start antihypertensive treatment in this patient?**


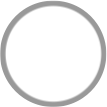
 Yes
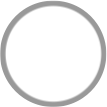
 No

* 18. **What would be your ideal treatment goal for this patient’s systolic blood pressure (in mmHg)?**


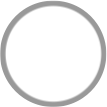
 <120
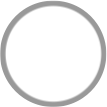
 141-150
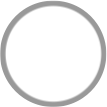
 >170

-
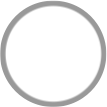
 120-130
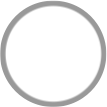
 151-160


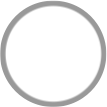
 131-140
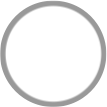
 161-170

**Antihypertensive treatment variation in very elderly**Case 6

*** 19. 
Female patient, 83 years, living in a residence with daily personal care**

**Findings**
- Systolic blood pressure 160 mmHg
- Muscle weakness 
- Slow gait speed 
- Stroke 2 years ago

**Your assessment**- Secondary preventive strategy for hypertension is advised according to current guidelines
- You consider this patient to be frail

**Do you start antihypertensive treatment in this patient?**


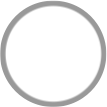
 Yes
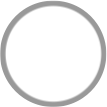
 No

* 20. **What would be your ideal treatment goal for this patient’s systolic blood pressure (in mmHg)?**


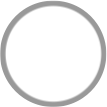
 <120
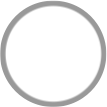
 141-150
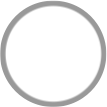
 >170

-
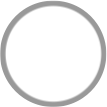
 120-130
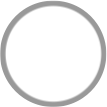
 151-160


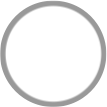
 131-140
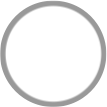
 161-170

**Antihypertensive treatment variation in very elderly**Case 7

*** 21. 
Female patient, 83 years, living in a residence with daily personal care**

**Findings**
- Systolic blood pressure 140 mmHg
- Unintentional weight loss 
- Myocardial infarction 2 years ago

**Your assessment**- Secondary preventive strategy for hypertension is advised according to current guidelines
- You don’t consider this patient to be frail

**Do you start antihypertensive treatment in this patient?**


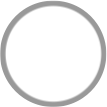
 Yes
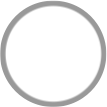
 No

* 22. **What would be your ideal treatment goal for this patient’s systolic blood pressure (in mmHg)?**


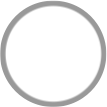
 <120
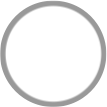
 141-150
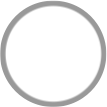
 >170

-
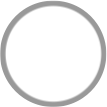
 120-130
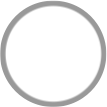
 151-160


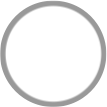
 131-140
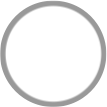
 161-170

**Antihypertensive treatment variation in very elderly**Case 8

*** 23. 
Male patient, 82 years, independent living with spouse, some help in housekeeping**

**Findings**
- Systolic blood pressure 140 mmHg
- Unintentional weight loss 
- Low level of activity 
- Myocardial infarction 2 years ago

**Your assessment**- Secondary preventive strategy for hypertension is advised according to current guidelines
- You consider this patient to be frail

**Do you start antihypertensive treatment in this patient?**


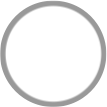
 Yes
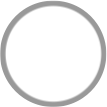
 No

* 24. **What would be your ideal treatment goal for this patient’s systolic blood pressure (in mmHg)?**


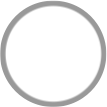
 <120
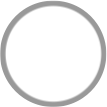
 141-150
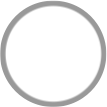
 >170

-
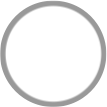
 120-130
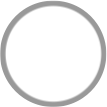
 151-160


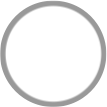
 131-140
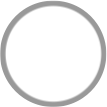
 161-170

**Antihypertensive treatment variation in very elderly**Final remarks

25. In a frail and multimorbid patient aged >80 years which of the reasons below would you find valid to stop antihypertensive treatment? (please select all the reasons you find valid)


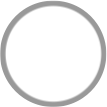
 Systolic blood pressure <120 mmHg


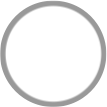
 Systolic blood pressure <140 mmHg


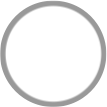
 Patient reports dizziness, when standing up


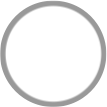
 Patient reports cognitive decline


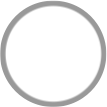
 Life expectancy, less than one year


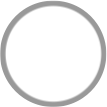
 Low estimated benefit (e.g. low estimated rate of stroke reduction)


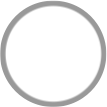
 Others, please specify:

26. Please state your email address if you give us permission to email you again. We plan to conduct additional studies (e.g. stopping statin, or other questions) 
It would be great to have you on board, again!

27. Please give us your feedback or any comments on this survey.
