# Supplementary material for: Variation in GP decisions on antihypertensive treatment in oldest-old and frail individuals across 29 countries
Source: BMC Geriatr. 2017 Apr 20;17:93. doi: 10.1186/s12877-017-0486-4 (PMC5399328; doi:10.1186/s12877-017-0486-4)
Supplement: Supplementary file 2 — Characteristics of the eight case vignettes used in this survey. (DOCX 18 kb) [file 12877_2017_486_MOESM2_ESM.docx]

**Additional file 2.** Characteristics of the eight case vignettes used in this survey_._

| Case | Frailty | Cardiovascular  disease | Systolic blood  pressure (mmHg) |
| --- | --- | --- | --- |
| 1 | No | No | 140 |
| 2 | Yes | No | 140 |
| 3 | No | No | 160 |
| 4 | Yes | No | 160 |
| 5 | No | Yes | 160 |
| 6 | Yes | Yes | 160 |
| 7 | No | Yes | 140 |
| 8 | Yes | Yes | 140 |

All patients were aged >80 years and presented at the GP’s office for routine control. None of the patients had blood pressure-related complaints and none was receiving any antihypertensive treatment.
