# Supplementary material for: Variation in GP decisions on antihypertensive treatment in oldest-old and frail individuals across 29 countries
Source: BMC Geriatr. 2017 Apr 20;17:93. doi: 10.1186/s12877-017-0486-4 (PMC5399328; doi:10.1186/s12877-017-0486-4)
Supplement: Supplementary file 3 — Participating countries: number of invited GPs and response rates per country. (DOCX 21 kb) [file 12877_2017_486_MOESM3_ESM.docx]

**Additional file 3.** Participating countries: number of invited GPs and response rates per country.

| Country | Invited (n=13,671) | Participated (n=2,543) | Response rate (%) |
| --- | --- | --- | --- |
| Austria | 549 | 28 | 5 |
| Bosnia Herzegovina | 260 | 26 | 10 |
| Brazil | 67 | 63 | 94 |
| Czech Republic | 356 | 27 | 8 |
| Denmark | 203 | 22 | 11 |
| Finland | 118 | 24 | 20 |
| France | 150 | 63 | 42 |
| Germany | 300 | 29 | 10 |
| Greece | 89 | 23 | 26 |
| Hungary | 515 | 332 | 64 |
| Ireland | 2576 | 401 | 16 |
| Israel | 395 | 140 | 35 |
| Italy | 120 | 38 | 32 |
| Latvia | 990 | 88 | 9 |
| Luxembourg | 40 | 7 | 18 |
| Macedonia | 28 | 21 | 75 |
| Netherlands | 1720 | 239 | 14 |
| New Zealand | 1524 | 39 | 3 |
| Norway | 99 | 31 | 31 |
| Poland | 79 | 69 | 87 |
| Portugal | 82 | 51 | 62 |
| Romania | 53 | 45 | 85 |
| Slovenia | 312 | 24 | 8 |
| Spain | 411 | 57 | 14 |
| Sweden | 130 | 34 | 26 |
| Switzerland | 1756 | 510 | 29 |
| Turkey | 648 | 17 | 3 |
| Ukraine | 73 | 69 | 95 |
| United Kingdom | 28 | 26 | 93 |
| Median (IQR) |  |  | **26 (10-62)** |
